# The Phenolic Fraction of *Mentha haplocalyx* and Its Constituent Linarin Ameliorate Inflammatory Response through Inactivation of NF-κB and MAPKs in Lipopolysaccharide-Induced RAW264.7 Cells

**DOI:** 10.3390/molecules22050811

**Published:** 2017-05-16

**Authors:** Xiangyang Chen, Shujing Zhang, Zinan Xuan, Dongyu Ge, Xiaoming Chen, Junjie Zhang, Qian Wang, Ying Wu, Bin Liu

**Affiliations:** 1 Department of Traditional Chinese Medicine Chemistry, School of Chinese Materia Medica, Beijing University of Chinese Medicine, Beijing 100102, China; chenxiangyang92@163.com; 2 Department of Scientific Research Center, School of Chinese Medicine, Beijing University of Chinese Medicine, Beijing 100029, China; jingshuzhang@126.com (S.Z.); gedongyu@sohu.com (D.G.); 3 Department of Microbiology and Immunology, School of Life Science, Beijing University of Chinese Medicine, Beijing 100029, China; thehappyworld@126.com (Z.X.); cxm2010z@163.com (X.C.); zhangjunjiebucm@163.com (J.Z.); 4 Department of Pathology, School of Chinese Medicine, Beijing University of Chinese Medicine, Beijing 100029, China; wangqianchai@163.com

**Keywords:** *Mentha haplocalyx*, phenolic fraction, linarin, anti-inflammation, NF-κB, MAPK, Akt

## Abstract

*Mentha haplocalyx* has been widely used for its flavoring and medicinal properties and as a traditional Chinese medicine with its anti-inflammation properties. The present study was designed to investigate the anti-inflammatory effects and potential molecular mechanisms of the phenolic fraction of *M. haplocalyx* (MHP) and its constituent linarin in lipopolysaccharide (LPS)-induced RAW264.7 cells. The high-performance liquid chromatography coupled with linear ion trap-orbitrap mass spectrometry (HPLC-LTQ-Orbitrap MS) was used to analyze the chemical composition of MHP. Using the enzyme-linked immunosorbent assay (ELISA) and quantitative realtime polymerase chain reaction (qRT-PCR), the expression of pro-inflammatory meditators and cytokines was measured at the transcriptional and translational levels. Western blot analysis was used to further investigate changes in the nuclear factor kappa B (NF-κB), mitogen-activated protein kinase (MAPK), and Akt signaling pathways. Fourteen phenolic constituents were identified from MHP based on the data of the mass spectrometry (MS)/MS analysis. MHP and linarin decreased the production of NO, tumor necrosis factor-α (TNF-α), interlenkin-1β (IL-1β), and IL-6. The messenger ribonucleic acid (mRNA) expression levels of inducible NO synthase (iNOS), TNF-α, IL-1β, and IL-6 were also suppressed by MHP and linarin. Further investigation showed that MHP and linarin down-regulated LPS-induced phosphorylation content of NF-κB p65, inhibitor kappa B α (IκBα), extracellular signal-regulated kinase (ERK), c-Jun NH_2_-terminal kinase (JNK), and p38. However, MHP and linarin showed no inhibitory effect on the phosphorylated Akt. These results suggested that MHP and linarin exerted a potent inhibitory effect on pro-inflammatory meditator and cytokines production via the inactivation of NF-κB and MAPKs, and they may serve as potential modulatory agents for the prevention and treatment of inflammatory diseases.

## 1. Introduction

Inflammation is an important defense mechanism of the host against invasive stimuli [1,2]. However, excessive inflammatory responses may give rise to various pathological changes related to human diseases, including asthma, rheumatoid arthritis, atherosclerosis, and cancer [3]. Many risk factors are responsible for inflammation, such as pathogens, impaired cells and tissues, and physical and chemical stimuli [4]. Macrophages are phagocytic white blood cells that play an important role in innate immune inflammatory responses. Activated macrophages secrete various cytokines such as tumor necrosis factor-α (TNF-α), interlenkin-1β (IL-1β), interlenkin-6 (IL-6), and other inflammatory cytokines [5], which show pleiotropic effects via activating adaptive immune responses and worsening inflammatory responses [6]. The nuclear factor kappa-B (NF-κB) signaling pathway is involved in regulating diverse gene expression during inflammatory responses [7]. When activated with extracellular stimuli, the NF-κB released from the cytoplasmic NF-κB-inhibitor kappa B (IκB) complex by the ubiquitination and degradation of IκB translocates into the nucleus [8,9], combining with specific DNA sequences and then regulating the transcription of inflammatory cytokines and mediators [10,11]. Moreover, phosphoinositide 3-kinase (PI3K)/Akt and mitogen-activated protein kinases (MAPKs) including extracellular signal-regulated kinase (ERK), p38, and c-Jun NH_2_-terminal kinase (JNK) subfamilies could activate NF-κB and regulate inflammatory responses [12,13].

*Mentha* species are widely used throughout the world as flavoring agents and medicinal plants, particularly for the treatment of common fever, cold, flu, and motion sickness [14,15]. Although the genus *Mentha* comprises more than 20 species, only the fresh aerial part of *Mentha haplocalyx* Briq. is described and used as a traditional Chinese herb in Chinese pharmacopoeia. The leaves of *M. haplocalyx* Briq. are also used in teas, beverages, jellies, syrups, candies, and other products. Modern pharmacological studies have revealed that *M. haplocalyx* shows various biological activities, such as antiallergenic, antimicrobial, anti-inflammatory, antioxidant, antitumor, antiviral, gastrointestinal protective, hepatoprotective, and chemopreventive activities [16]. Other well-known *Mentha* species, including *M. spicata*, *M. longifolia*, *M. piperita*, *M. pulegium*, and *M. arvensis*, have been reported to express similar biological activities. For instance, gastrointestinal protective properties have been reported for *M. longifolia*, *M. pulegium*, and *M. arvensis* [17,18,19], chemopreventive activities for *M. piperita* and *M. spicata* [20,21], and antiviral effects for *M. piperita*, *M. spicata*, and *M. longifolia* [22,23]. *Mentha* species are characterized by two important classes of secondary metabolites: essential oils and phenolic compounds [24]. Numerous researchers have proved that these compounds contribute to the multiple biological activities of *Mentha* species [24,25].

In traditional Chinese medicine, *M. haplocalyx* is used frequently as a herb that releases the “exterior” in the common cold of wind-heat type, headache, red eye, measles, and thoracic and abdominal oppression [26]. Pharmacological studies have revealed that “exterior releasing herbs” are effective against viral and bacterial infections that usually induce inflammation [27]. Based on the fact that heat is related to inflammation in Chinese medicine [28] and that *M. haplocalyx* has cooling properties, it can be assumed that *M. haplocalyx* could exert an inhibitory effect on inflammation. Indeed, some studies have proved that flavonoids and phenolic acids from the aqueous extract of *M. haplocalyx* [29,30,31] express high anti-inflammatory activity [16,32]. In our previous work, we have optimized a method for the purification of the phenolic fraction of *M. haplocalyx* (MHP), mainly consisting of flavonoids and phenolic acids [33]. In the present study, our particular interest was to investigate the mechanism of the anti-inflammatory effect of *M. haplocalyx* extract, as well as to analyze the chemical composition of MHP by high-performance liquid chromatography coupled with linear ion trap-orbitrap mass spectrometry (HPLC-LTQ-Orbitrap MS). We explored the mechanism of inflammatory activity by evaluating the impact of MHP on the production of pro-inflammatory meditators and the activation of NF-κB, Akt, and MAPKs in lipopolysaccharide (LPS)-induced RAW264.7 cells. Since the linarin was the main flavonoid from MHP, the mechanism of its activity was also investigated for the first time in this study.

## 2. Results

### 2.1. Analysis of the Chemical Composition of the Phenolic Fraction of Mentha haplocalyx by HPLC-LTQ-Orbitrap MS

**Figure 1:**
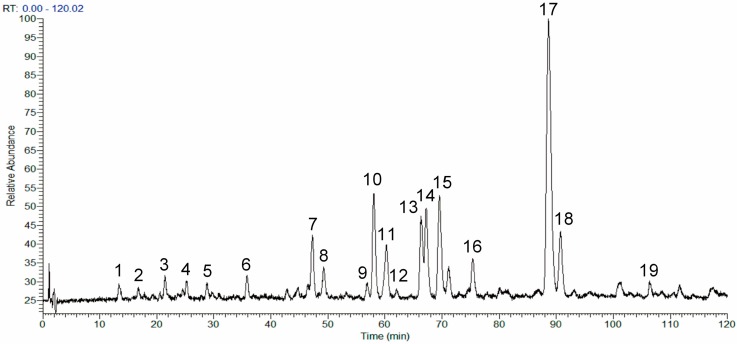
Total ion chromatogram profile of the phenolic fraction of *Mentha haplocalyx* (MHP).

Structural elucidation of the MHP constituents was performed according to the obtained chromatographic retention times and mass spectrometry (MS)/MS data by comparison with the data of available reference compounds including rosmarinic acid, linarin, or literature data. As a result, 14 phenolic compounds were identified in the MHP sample. The total ion chromatogram (TIC) profile of MHP is shown in Figure 1 and the data for compound identification are summarized in Table 1.

### 2.2. Effects of the Phenolic Fraction of Mentha haplocalyx and Linarin on NO Production and iNOS Expression Level in LPS-Induced RAW264.7 Cells

**Figure 2:**
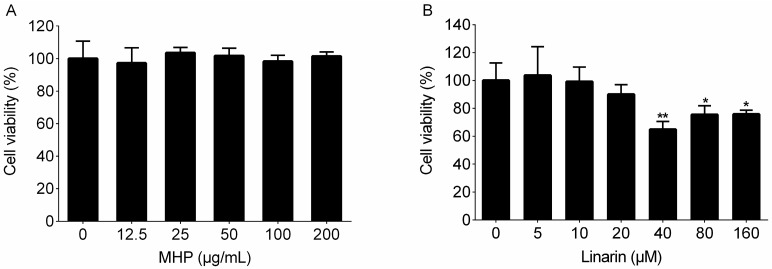
Effects of MHP and linarin on the viability of RAW264.7 cells. (**A**) The cells were treated with MHP (0, 12.5, 25, 50, 100, and 200 μg/mL) for 24 h; (**B**) The cells were treated with linarin (0, 5, 10, 20, 40, 80, and 160 μM) for 24 h. Cell viability was determined by the 3-(4,5-dimethylthiazol-2-yl)-2,5-diphenyltetrazolium bromide (MTT) assay. The data shown are representative of three experiments and expressed as mean ± SD. * *p* < 0.05 and ** *p* < 0.01 versus the normal control group.

The toxicity of drugs could have an influence on the proliferation and function of cells, misleading the judgement of their efficacy. Thus, we firstly evaluated the cell cytotoxicity of MHP and linarin. The RAW264.7 cells were treated with increased concentrations of MHP and linarin for 24 h and cell cytotoxicity was examined by the 3-(4,5-dimethylthiazol-2-yl)-2,5-diphenyltetrazolium bromide (MTT) assay. As shown in Figure 2, MHP and linarin did not exhibit toxic effects on RAW264.7 cells at 0–200 μg/mL and 0–20 μM, respectively. 

**Figure 3:**
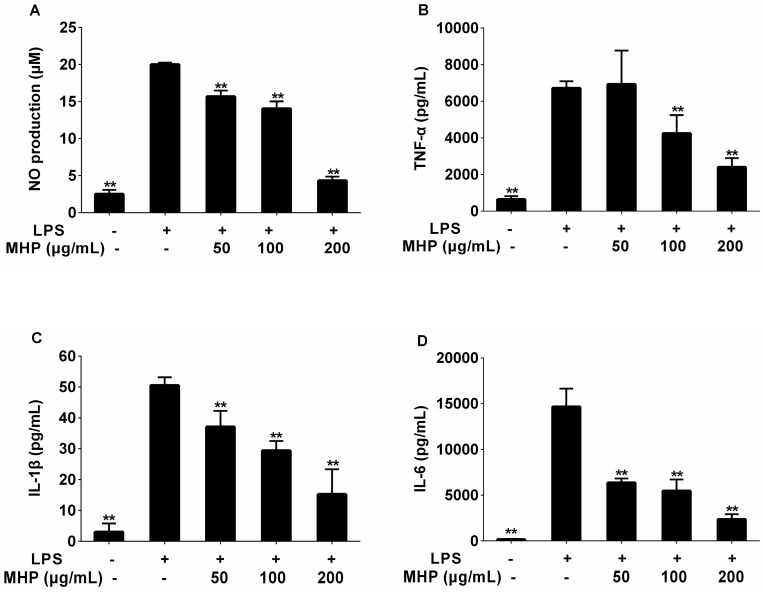
Effect of MHP on the production of nitric oxide (NO), tumor necrosis factor-α (TNF-α), interlenkin-1β (IL-1β), and IL-6 in LPS-induced RAW264.7 cells. The cells were pretreated with MHP (50, 100, and 200 μg/mL) for 1 h, and then were stimulated with LPS (1.0 μg/mL) for 24 h. The level of NO (**A**) in the supernatant was determined using the nitric oxide assay kit. The levels of TNF-α (**B**), IL-1β (**C**), and IL-6 (**D**) in the supernatant were assayed using enzyme-linked immunosorbent assay (ELISA) kits. The data shown are representative of three experiments and expressed as mean ± SD. ** *p* < 0.01 versus the group treated with LPS alone.

**Figure 4:**
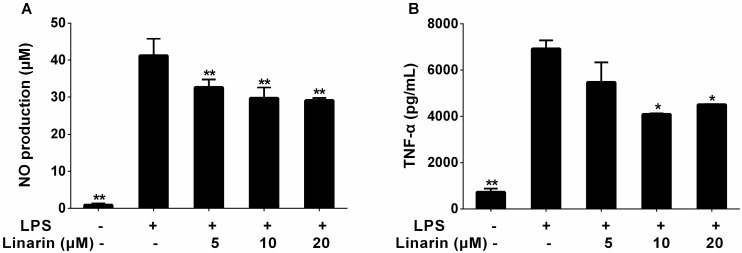

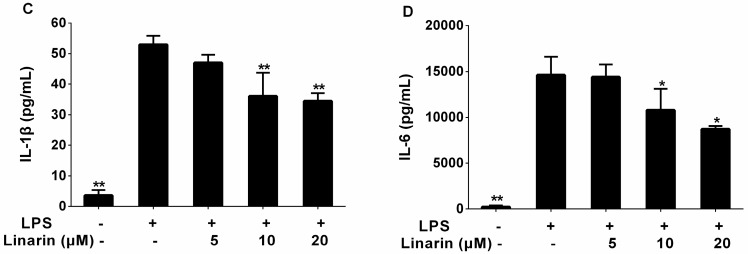
Effect of linarin on the production of NO, TNF-α, IL-1β and IL-6 in LPS-induced RAW264.7 cells. The cells were pretreated with linarin (5, 10, and 20 μM) for 1 h, and then were stimulated with LPS (1.0 μg/mL) for 24 h. The level of NO (**A**) in the supernatant was determined using the nitric oxide assay kit. The levels of TNF-α (**B**), IL-1β (**C**), and IL-6 (**D**) in the supernatant were assayed using ELISA kits. The data shown are representative of three experiments and expressed as mean ± SD. * *p* < 0.05 and ** *p* < 0.01 versus the group treated with LPS alone.

**Figure 5:**
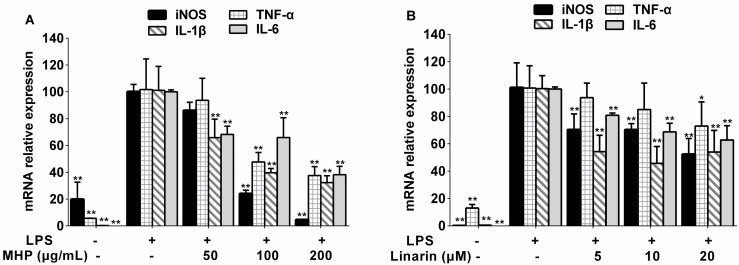
Effects of MHP and linarin on the mRNA expression of inducible NO synthase (iNOS), TNF-α, IL-1β, and IL-6 in LPS-induced RAW264.7 cells. (**A**) The cells were pretreated with MHP (50, 100, and 200 μg/mL) for 1 h, and then were stimulated with LPS (1.0 μg/mL) for 24 h. (**B**) The cells were pretreated with linarin (5, 10, and 20 μM) for 1 h, and then were stimulated with LPS (1.0 μg/mL) for 24 h. The mRNA expression levels of iNOS, TNF-α, IL-1β, and IL-6 were measured by quantitative real time polymerase chain reaction (qRT-PCR). Glyceraldehyde 3-phosphate dehydrogenase (GAPDH) was used as the internal control. The data shown are representative of three experiments and expressed as mean ± SD. * *p* < 0.05 and ** *p* < 0.01 versus the group treated with LPS alone.

To investigate the anti-inflammatory activities of MHP and linarin, we studied the inhibitory effect of MHP and linarin on LPS-induced nitric oxide (NO) production in RAW264.7 cells. As shown in Figure 3A and Figure 4A, the NO production level significantly increased after LPS-stimulation for 24 h in RAW264.7 cells. MHP at 50–200 μg/mL and linarin at 5–20 μM showed markedly inhibitory effects on the increase of NO in a dose-dependent manner. Subsequently, we investigated the cause of reduced NO production by measuring the expression of inducible NO synthase (iNOS) mRNA. Quantitative real-time polymerase chain reaction (qRT-PCR) analysis showed that both MHP and linarin were able to inhibit the expression of iNOS mRNA (Figure 5A,B). The results suggested that MHP and linarin inhibited NO production by down-regulating the expression of iNOS mRNA.

### 2.3. Effects of the Phenolic Fraction of Mentha haplocalyx and Linarin on the Production of TNF-α, IL-1β, and IL-6 in LPS-Induced RAW264.7 Cells

To determine the effects of MHP and linarin on the production of inflammatory cytokines including TNF-α, IL-1β, and IL-6, the RAW264.7 cells were pretreated with LPS in the presence or absence of screened concentrations of MHP and linarin. The results of enzyme-linked immunosorbent assay (ELISA) showed that the amount of TNF-α, IL-1β, and IL-6 significantly increased in RAW264.7 cells induced by LPS alone. As shown in Figure 3B–D, the level of TNF-α was significantly inhibited by MHP at 100 and 200 μg/mL, while the levels of IL-1β and IL-6 were markedly suppressed by MHP at 50, 100, and 200 μg/mL. Meanwhile, linarin remarkably suppressed the secretion of TNF-α, IL-1β, and IL-6 at concentrations of 10 and 20 μM (Figure 4B–D).

### 2.4. Effects of the Phenolic Fraction of Mentha haplocalyx and Linarin on TNF-α, IL-1β, and IL-6 mRNA Expression in LPS-Induced RAW264.7 Cells

In order to investigate whether MHP and linarin inhibited the expression of pro-inflammatory cytokines at the transcriptional level, we examined TNF-α, IL-1β, and IL-6 mRNA expression levels in RAW264.7 cells. As expected in Figure 5A, 100 and 200 μg/mL of MHP significantly inhibited the LPS-induced increase of the TNF-α mRNA expression while MHP at 50, 100, and 200 μg/mL decreased the expression of IL-1β and IL-6 mRNA induced by LPS. In addition, linarin significantly repressed IL-1β and IL-6 mRNA expression at 5–20 μM and the mRNA expression level of TNF-α was significantly down-regulated by linarin at 20 μM (Figure 5B).

### 2.5. Effects of the Phenolic Fraction of Mentha haplocalyx and Linarin on NF-κB p65 and IκBα in LPS-Induced RAW264.7 Cells

**Figure 6:**
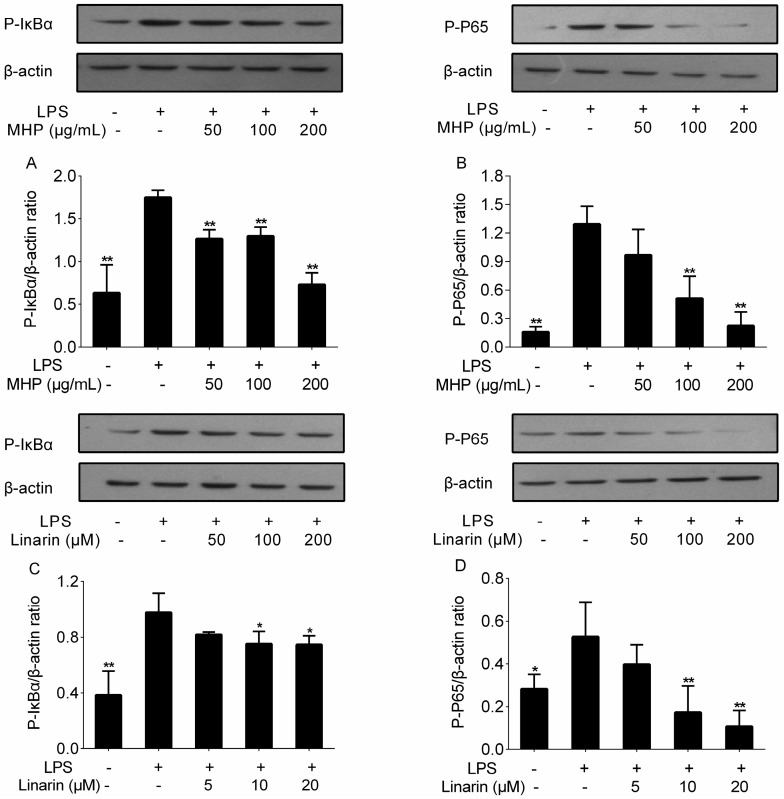
Effects of MHP and linarin on the nuclear factor kappa B (NF-κB) signaling pathway in LPS-induced RAW264.7 cells. (**A**,**B**) The cells were pretreated with MHP (50, 100, and 200 μg/mL) for 4 h, and then were stimulated with LPS (1.0 μg/mL) for 30 min; (**C**,**D**) The cells were pretreated with linarin (5, 10, and 20 μM) for 4 h, and then were stimulated with LPS (1.0 μg/mL) for 30 min. The levels of phosphorylated inhibitor kappa B α (p-IκBα) and p-p65 were detected by Western blot analysis. β-actin was used as the internal control. The data shown are representative of three experiments and expressed as mean ± SD. * *p* <0.05 and ** *p* < 0.01 versus the group treated with LPS alone.

The activation of NF-κB in response to LPS is a vital step in inducing the expression of inflammatory cytokines, such as TNF-α, IL-1β, and IL-6 [34]. Here, we investigated whether MHP and linarin inhibited the phosphorylation of inhibitor kappa B α (IκBα) and p65. As depicted in Figure 6, the relative content of phosphorylated (p-)IκBα and p-p65 significantly increased after LPS stimulation, which suggested that LPS stimulation triggered the phosphorylation of IκBα and p65. However, these increases were inhibited by MHP and linarin. MHP treatment significantly restrained the level of p-IκBα at 50, 100, and 200 μg/mL (Figure 6A), and at 100 and 200 μg/mL for p-p65 (Figure 6B). The increased levels of p-p65 and p-IκBα were also repressed by linarin at 10 and 20 μM (Figure 6C,D). These results indicated that MHP and linarin exerted anti-inflammatory effects through inhibition of LPS-induced activation of the NF-κB signaling pathway.

### 2.6. Effects of the Phenolic Fraction of Mentha haplocalyx and Linarin on Akt in LPS-Induced RAW264.7 Cells

**Figure 7:**
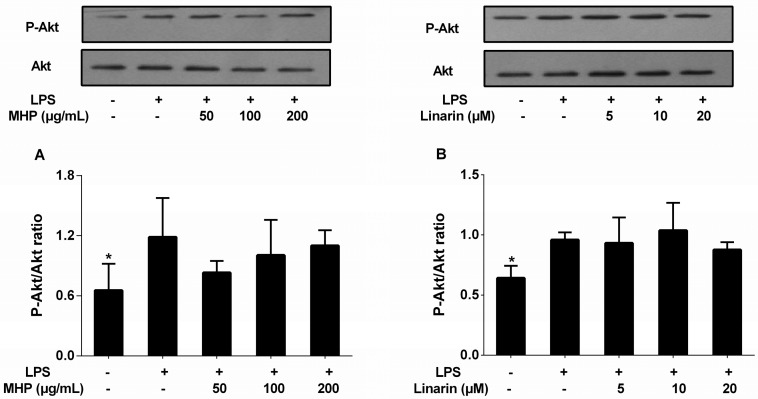
Effects of MHP and linarin on the Akt signaling pathway in LPS-induced RAW264.7 cells. (**A**) The cells were pretreated with MHP (50, 100, and 200 μg/mL) for 4 h, and then were stimulated with LPS (1.0 μg/mL) for 30 min; (**B**) The cells were pretreated with linarin (5, 10 and 20 μM) for 4 h, and then were stimulated with LPS (1.0 μg/mL) for 30 min. The levels of p-Akt and Akt were detected by Western blot analysis. The data shown are representative of three experiments and expressed as mean ± SD. * *p* < 0.05 versus the group treated with LPS alone.

A previous study has reported that activated Akt, a key protein of the PI3K signaling pathway, could turn on the activation of NF-κB [12]. Thus, we investigated the inhibitory effects of MHP and linarin on LPS-induced expression of phosphorylated Akt protein in RAW264.7 cells. However, the results shown in Figure 7A,B indicate that MHP and linarin exerted no inhibitory effect on the LPS-induced increase of the phosphorylation level of Akt.

### 2.7. Effects of the Phenolic Fraction of Mentha haplocalyx and Linarin on MAPKs in LPS-Induced RAW264.7 Cells

**Figure 8:**
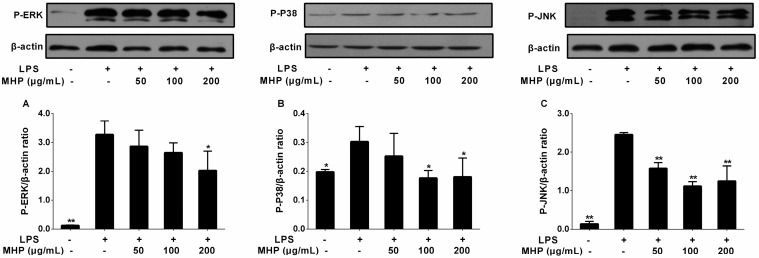

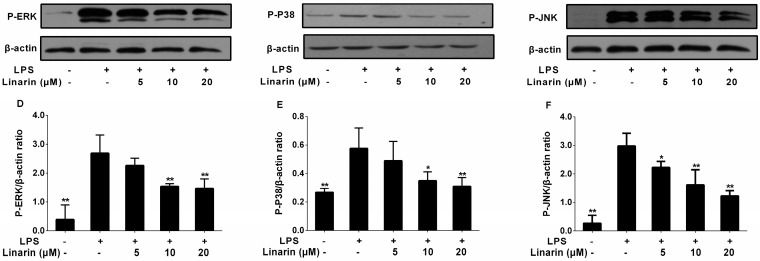
Effects of MHP and linarin on the mitogen-activated protein kinase (MAPK) signaling pathway in LPS-induced RAW264.7 cells. (**A**–**C**) The cells were pretreated with MHP (50, 100, and 200 μg/mL) for 4 h, and then were stimulated with LPS (1.0 μg/mL) for 30 min. (**D**–**F**) The cells were pretreated with linarin (5, 10, and 20 μM) for 4 h, and then were stimulated with LPS (1.0 μg/mL) for 30 min. The levels of the phosphorylated extracellular signal-regulated kinase (ERK), p38, and c-Jun NH2-terminal kinase (JNK) were detected by Western blot analysis. β-actin was used as the internal control. The data shown are representative of three experiments and expressed as mean ± SD. * *p* < 0.05 and ** *p* < 0.01 versus the group treated with LPS alone.

To further explore whether MHP and linarin exerted inhibitory effects on the inflammatory mediators and cytokines through inactivation of the MAPK signaling pathway, we investigated the phosphorylation levels of MAPKs including ERK1/2, JNK, and p38. As observed in Figure 8, LPS stimulation increased the relative content of p-p38, p-JNK, and p-ERK1/2. MHP treatment remarkably inhibited the phosphorylation level of JNK at 50, 100, and 200 μg/mL (Figure 8C) and suppressed the phosphorylation level of p38 at 100 and 200 μg/mL (Figure 8B), while at 200 μg/mL for the phosphorylated ERK (Figure 8A). Furthermore, linarin significantly inhibited the phosphorylation of JNK at 5, 10, and 20 μM (Figure 8F), but at 10 and 20 μM for the phosphorylation of ERK and p38 (Figure 8D,E). Analytical data indicated that MHP and linarin exerted an anti-inflammation effect via the inhibition of the LPS-induced MAPK signaling pathway. 

## 3. Discussion

Pharmacological investigations have shown that anti-inflammation is an essential feature of many *Mentha* species. *M. haplocalyx* ethanol extract significantly lessened the severity of airway inflammation by inhibiting the production of immunoglobulin E, IL-4, and IL-6 in bronchoalveolar lavage fluid and lung tissue [35]. Ethyl acetate and aqueous fractions of *M. spicata* effectively reduced the inflammation induced by carrageenan and cotton pellet [36]. The aqueous ethanol (70%) extract of *M. arvensis* at 100 μg/mL showed a strong inhibitory effect on IL-8 secretion [37], and the aqueous methanol (80%) extract of *M. arvensis*, the main constituents of which were linarin and rosmarinic acid, showed inhibitory activity against the strain *Chlamydia pneumoniae* CWL-029 [38]. The hexane fraction of *M. longifolia*, mainly containing phenolic and flavonoid compounds, was able to significantly reduce NO production, iNOS, and TNF-α mRNA expression at 0.05–0.20 mg/mL in LPS-induced J774A.1 cells [39]. The results of all the studies mentioned above suggest that *Mentha* species could serve as an important source of anti-inflammatory agents. 

In this paper, we investigated the chemical composition and anti-inflammatory activity of the phenolic fraction from *M. haplocalyx*. The chemical profile of the MHP sample was analyzed by HPLC-LTQ-Orbitrap MS and showed that its main constituents were phenolic acids and flavonoids, and some of them, such as protocatechuic aldehyde, caffeic acid, rosmarinic acid, salvianolic acid B, and diosmin, have been reported to display inhibitory effects on inflammation [40,41,42,43,44]. These bioactive constituents probably contribute to the anti-inflammatory activity of MHP. Flavonoid linarin is also one of the main constituents of the MHP extract. Previous research has shown that linarin from *M. arvensis* exhibited a selective inhibitory effect on acetylcholinesterase in a concentration-dependent manner [45]. It has also been reported that linarin possesses analgesic, antipyretic [46], and neuroprotective activities [47], and showed a protective effect against d-glactosamine/LPS-induced fulminant hepatic failure [48]. Furthermore, a tissue distribution study of linarin has revealed that it was rapidly and widely distributed in the heart, liver, spleen, lung, kidney, brain, muscle, stomach, small intestine, epididymis, pancreas, and bladder of rats after intragastric administration [49]. Since there was little data on the anti-inflammatory mechanism of linarin [50], the present study explored its involvement in the anti-inflammatory action of the MHP extract. 

NO is a small diffusible molecule, which exerts a regulating effect on various biological functions including apoptosis, neurotransmission, inflammation, and blood vessel tone [51]. NO is generated from l-arginine by three types of NO synthases including neuronal NOS (nNOS), iNOS, and endothelial NOS (eNOS) [51]. Among them, iNOS is the main enzyme to catalyze NO production in acute and chronic inflammation, and inflammatory stimuli such as LPS can cause its expression [7,52]. Our data indicated that both MHP and linarin significantly suppressed the LPS-induced NO production in RAW264.7 cells with a concentration-dependent manner via down-regulating the transcriptional level of iNOS.

Apart from NO, many pro-inflammatory cytokines including TNF-α, IL-6, and IL-1β are produced and take part in the process of inflammatory responses. TNF-α acts as a principal endogenous mediator during inflammatory responses and is also capable of stimulating the secretion of IL-6 and IL-1β, which could exert a synergistic effect with TNF-α [2,53], causing further damage to the body. Inhibiting the synthesis or release of these inflammatory cytokines could emerge as a potential therapeutic approach for inflammatory diseases. In the present study, MHP significantly blocked LPS-induced overproduction of TNF-α, IL-1β, and IL-6. Linarin attenuated TNF-α, IL-1β, and IL-6 production induced by LPS as well, which was consistent with the reported article [50]. Additionally, further investigation demonstrated that MHP and linarin treatment down-regulated the expression levels of TNF-α, IL-1β, and IL-6 mRNA. The above results indicated that MHP and linarin exerted an inhibitory effect on LPS-induced inflammation by suppressing the expression of inflammatory cytokines at the transcriptional and translational levels.

By modulating the secretion of inflammatory cytokines, NF-κB exerts a critical effect on regulating inflammatory responses to extracellular stimuli [34,54]. The major forms of NF-κB are composed of p50 and p65, which are bound to the cytoplasmic inhibitory protein IκB in the resting state [54]. Once activated by external stimuli, IκB is phosphorylated by IκB kinase (IKK) which leads to the degradation of IκBα [55], and p65 is then released and translocates to the nucleus where it may trigger the expression of multiple inflammatory genes. Among the identified constituents of MHP, protocatechuic aldehyde exerted an anti-inflammatory effect on myocardial ischemia/reperfusion injury through inhibiting the NF-κB pathway [40]. Caffeic acid exhibited the anti-inflammatory property via reducing the activation of NF-κB targeting genes [41]. Both salvianolic acid B and diosmin showed a protective effect on lung inflammation by suppressing the expression of TNF-α, IL-1β, and IL-6 through inactivation of the NF-κB pathway [42,43]. Therefore, MHP was expected to exert an anti-inflammatory effect by inhibiting the NF-κB pathway. Data in this paper showed that treatment with definite concentrations of MHP effectively blocked LPS-induced phosphorylation of IκBα and p65. Meanwhile, the increased phosphorylation levels of IκBα and p65 were also down-regulated by linarin. These suggested that MHP and linarin were able to repress the activation of the NF-κB pathway in LPS-induced RAW264.7 cells.

In addition to the NF-κB pathway, the PI3K/Akt and MAPK pathways play essential roles in regulating inflammatory cytokine production in macrophages. It has been reported that Akt and MAPKs were involved in the activation of NF-κB [56,57]. Akt is a key downsteam protein of PI3K and shows diverse roles in cellular growth, adhesion, and inflammation responses [58]. However, our finding showed that LPS-induced phosphorylated Akt was not blocked by MHP. Rosmarinic acid and linarin were two main constituents of MHP. In the previous study of the molecular mechanism of melanogenesis, rosmarinic acid did not reduce Akt phosphorylation in B16 melanoma cells [59]. The results of the present study also demonstrated that linarin displayed no significant inhibitory effect on the phosphorylated Akt. These results suggest that other signaling pathways may be involved in the anti-inflammatory mechanisms of MHP and linarin. Therefore, the impacts of MHP and linarin on MAPKs were further investigated. MAPKs including JNK, p38, and ERK are reported to be closely associated with diverse cellular processes, such as differentiation, proliferation, apoptosis, and immune responses [60]. In general, mitogen and differentiation signals are able to activate the ERK while stress stimuli activate p38 and JNK [61]. Many studies have illustrated that the up-regulation of TNF-α, IL-1β, and IL-6 is diminished by phytochemicals through the inhibition of the activation of ERK, JNK, and p38 [6,62]. In this paper, MHP and linarin down-regulated the phosphorylation levels of ERK, JNK, and p38. Taken together, these findings show that MHP and linarin could inhibit the activation of the NF-κB pathway via suppressing MAPKs activities. 

**Figure 9:**
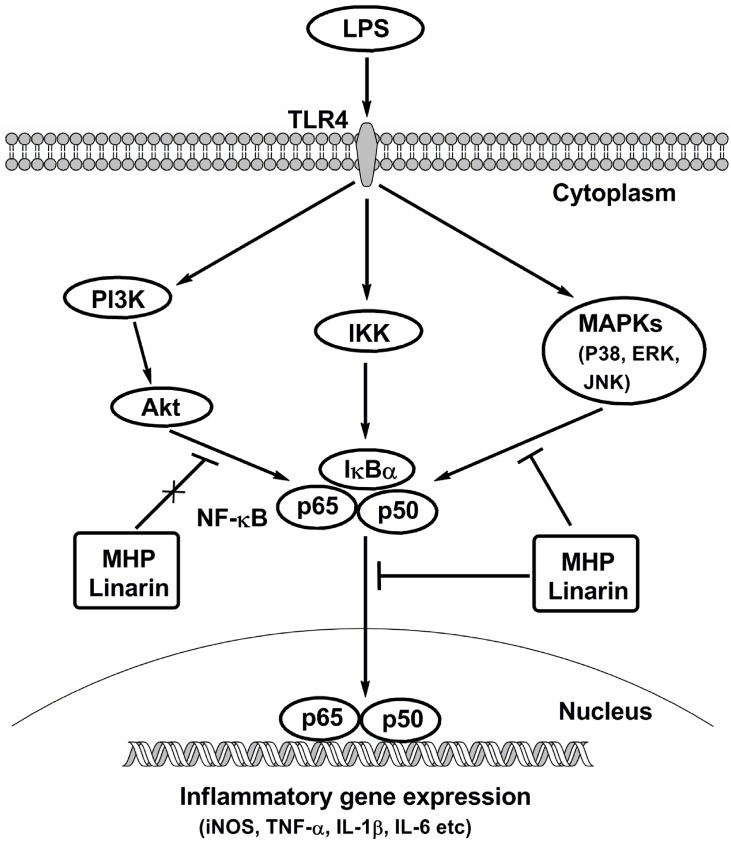
Possible roles of MHP and linarin in LPS-induced inflammatory responses in RAW264.7 cells. TLR4: Toll-like receptor 4; IKK: IκB kinase; PI3K: phosphoinositide 3-kinase.

In conclusion, the present study revealed that the phenolic fraction of *M. haplocalyx* and linarin showed potent inhibitory effects on the secretion of NO, TNF-α, IL-1β, and IL-6 and the mRNA expression of iNOS, TNF-α, IL-1β, and IL-6 in LPS-induced RAW264.7 cells. As shown in Figure 9, these effects were closely related to the inhibition of LPS-induced activation of NF-κB and MAPKs signals. The results not only clarified the anti-inflammatory molecular mechanisms of MHP and linarin, but also pointed out the potential value of MHP and linarin as anti-inflammatory agents for the prevention and treatment of inflammatory diseases.

## 4. Materials and Methods

### 4.1. Reagents

Linarin and rosmarinic acid were purchased from Must Bio-technology Co. (Chengdu, China). Lipopolysaccharides and 3-(4,5-dimethylthiazol-2-yl)-2,5-diphenyl tetrazolium bromide (MTT) were purchased from Sigma-Aldrich, Inc. (St. Louis, MO, USA). Dulbecco’s modified eagle’s medium (DMEM) and fetal bovine serum (FBS) were purchased from Corning Inc. (Corning, NY, USA). Nitric oxide assay kit was purchased from Beyotime Biotech. (Jiangsu, China). Mouse TNF-α, IL-1β, and IL-6 ELISA kits were purchased from eBioscience, Inc. (San Diego, CA, USA). Trizol, RevertAid™ first strand cDNA synthesis kit, power sybr green pcr master mix, and Super Signal^TM^ West Pico Chemiluminescent Substrate were purchased from Thermo Fisher Scientific Inc. (Waltham, MA, USA). Oligonucleotide primers were synthesized by Sangon Biotech Co., Ltd. (Shanghai, China). Horse-radish peroxidase-conjugated anti-rabbit or anti-mouse IgG secondary antibodies were obtained from Cell Signaling Technology (Danvers, CO, USA). Fluorescein-conjugated affinipure goat anti-rabbit IgG was purchased from Zhongshan Golden Bridge Biotechnology Co., Ltd. (Beijing, China). Antibodies against phospho (p)-ERK, p-p38, p-JNK, p-Akt, Akt, p-p65, and p-IκBα were obtained from Cell Signaling Technology. All other reagents were of analytical grade.

### 4.2. Preparation of the Phenolic Fraction of Mentha haplocalyx Extract

The sample (300 g) of *M. haplocalyx* was extracted three times by refluxing with 4800 mL of 30% ethanol for 1.5 h each time. Then the extracted solution was filtered and the procedure was repeated another two times. The filtered extracts were mixed together and then concentrated until there was no alcoholic odour. The concentrate was centrifuged at 3000 rpm for 30 min. The pH of the supernatant collected was adjusted to 3 by adding acetic acid. The sample solution was applied onto a column of treated HPD-400 macroporous resin (Cangzhou Baoen Chemical Co., Ltd., Cangzhou, Hebei, China). The elution conditions were as follows: 20% ethanol for two column volume, 70% ethanol for three column volume. The eluent of 70% ethanol was collected and then concentrated to dryness. MHP was obtained after the residue was dried under reduced pressure. For in vitro experiments, the phenolic fraction was dissolved in dimethyl sulfoxide with a final concentration of less than 0.1%. 

### 4.3. HPLC-MS/MS Analysis of MHP

MHP (10 mg) was dissolved using 70% methanol in a 25 mL volumetric flask, and the solution was filtered by a 0.45 μM microporous membrane.

HPLC-MS/MS analysis was performed using a LTQ-Orbitrap XL mass spectrometer (Thermo Scientific, Bremen, Germany). Analysis was carried out at 30 °C on an Inersil C18 column (4.6 mm × 150 mm, Waters, Milford, MA, USA). The mobile phase system was composed of acetonitrile (A) and 0.3% formic acid aqueous solution (B). A gradient program was used as follows: 0–40 min, 5%–15% A; 40–70 min, 15%–19.5% A; 70–90 min, 19.5%–22% A; 90–120 min, 22%–30% A. The flow rate was kept at 1 mL/min with a post-column splitting ratio of 1:4 and the sample volume injected was set at 5 μL.

The operating conditions of mass spectrometry were as follows: electrospray ionization (ESI) source in positive or negative mode, capillary temperature of 350 °C; sheath gas flow, 30 arb; auxiliary gas flow, 10 arb; positive mode, 4 kV of spray voltage, 25 V of capillary voltage; 110 V of tube lens negative mode, 3 kV of spray voltage, −35 V of capillary voltage; −110 V of tube lens. The range of full-scan mass was from *m*/*z* 100 to 1000. The resolution of the Orbitrap analyzer was set at 30,000. The three most intense ions from the one-stage mass spectrometry scanning were selected for further MS^2^ analysis. The other specific parameters were as follows: collision-induced dissociation (CID) of activation type, 2 amu of isolation width, 30 ms of activation time, 35% of normalized collision energy.

### 4.4. Cell Culture Conditions and Treatment

The RAW264.7 macrophage cell line was purchased from China Infrastructure of Cell Line Resource (Beijing, China). RAW264.7 cells were cultured in DMEM supplemented with 10% FBS and 1% penicillin-streptomycin in a humidified incubator with 5% CO_2_ at 37 °C. The cells were treated with MHP and linarin at different concentrations and then stimulated with LPS (1 μg/mL) for the incubation time.

### 4.5. Cell Viability Assay

The RAW264.7 cells were seeded in 96-well plates overnight, followed by incubation with MHP (0, 12.5, 25, 50, 100, and 200 μg/mL) and linarin (0, 5, 10, 20, 40, 80, and 160 μM) for 24 h. After treatment for 24 h, the supernatant was removed, and the MTT solution (10 μL) and fresh culture medium (90 μL) were added to each well. The cells were incubated for 4 h, after which the culture medium was removed and DMSO was added to dissolve the crystals for an additional 10 min. The value of optical density was read on a microplate reader (MK3, Thermoelectric Technology Instrument Co., Ltd., Shanghai, China) at 492 nm.

### 4.6. Effect on NO Production

The RAW264.7 cells were seeded in 96-well plates overnight and then treated with MHP (50, 100, and 200 μg/mL) and linarin (5, 10, and 20 μM) for 1 h, followed by incubation with LPS (1 μg/mL) for an additional 24 h. Griess reagent was used to determine the content of nitric oxide in the cell culture supernatant. Briefly, the culture supernatant (50 μL) was reacted with an equal volume of Griess reagent (50 μL), and the optical density of each well was measured on the microplate reader at 570 nm.

### 4.7. Cytokines Measurement

For the measurement of TNF-α, IL-1β, and IL-6, the RAW264.7 cells were seeded in 24-well plates overnight and then treated with MHP (50, 100, and 200 μg/mL) and linarin (5, 10, and 20 μM) for 1 h, followed by incubation with LPS (1 μg/mL) for an additional 24 h. The content of the pro-inflammatory cytokines including TNF-α, IL-1β, and IL-6 in the supernatant were assayed using the ELISA kits according to the manufacturer’s instructions.

### 4.8. RNA Extraction and qRT-PCR

Total RNA was isolated using the Trizol reagent according to the manufacturer’s protocol. Reverse transcription of total RNA (3.0 μg) was performed by using the RevertAid™ first strand cDNA synthesis kit on a MyCycler Thermal Cycler (Bio-Rad, Hercules, CA, USA). qRT-PCR was implemented using the SYBR qPCR kit with 2 μL cDNA on a StepOnePlus™ Real-Time PCR System (Applied Biosystems, Foster City, CA, USA). The thermal cycling conditions were as follows: 95 °C for 10 min, and then 40 cycles of 95 °C for 15 s, 60 °C for 1 min, and finally 95 °C for 15 s and 60 °C for 1 min. The mRNA expression levels of iNOS, TNF-α, IL-1β, and IL-6 were normalized to GAPDH. The following primers were used for PCR amplification: iNOS, 5′-GGTGAAGGGACTGAGCTGTT-3′ (forward) and 5′-ACGTTCTCCGTTCTCTTGCAG-3′ (reverse); TNF-α, 5′-TATGGCTCAGGGTCCAACTC-3′ (forward) and 5′-GGAAAGCCCATTTGAGTCCT-3′ (reverse); IL-1β, 5′-CTCACAAGCAGAGCACAAGC-3′ (forward) and 5′-CAGTCCAGCCCATACTTTAGG-3′ (reverse); IL-6, 5′-CGGAGAGGAGACTTCACAGAG-3′ (forward) and 5′-CATTTCCACGATTTCCCAGA-3′ (reverse); GAPDH, 5′-GGTTGTCTCCTGCGACTTCA-3′ (forward) and 5′-TGGTCCAGGGTTTCTTACTCC-3′ (reverse).

### 4.9. Western Blot Analysis

The RAW264.7 cells were seeded in 100-mm dishes overnight, after which they were treated with MHP (50, 100, and 200 μg/mL) and linarin (5, 10, and 20 μM) for 4 h, and further incubated with LPS (1 μg/mL) for an additional 30 min. Subsequently, the cells were rinsed two times with cold phosphate-buffered saline (PBS), lysed using ice-cold radioimmunoprecipitation assay (RIPA) buffer supplemented with a cocktail of protease and phosphatase inhibitors, and placed on ice for 30 min. The supernatants were collected after centrifuging at 10,000 g for 15 min at 4 °C. The bicinchoninic acid (BCA) protein assay kit was used to measure protein content for each sample. Protein samples were separated on a 10% SDS-PAGE using PowerPac™ Basic Power Supply (Bio-Rad, Hercules, CA, USA), blotted onto PVDF membranes and then blocked for 1 h at room temperature with 5% nonfat dry milk in Tris-buffered saline with Tween 20 (TBST) solution. The membranes were then incubated at 4 °C overnight with 1:1000 dilutions of the primary antibodies. After washed with TBST solution three times for 10 min, the membranes were incubated with HRP-conjugated secondary antibody for 1 h at room temperature. Finally, the membranes were washed with the TBST solution three times for 10 min and chemiluminescent signals of the protein bands were developed using the Super Signal^T^^M^ West Pico Chemiluminescent Substrate (Thermo Fisher Scientific Inc.).

### 4.10. Statistical Analysis

The results were expressed as the means ± standard deviation (SD). Multiple group comparisons were performed using one-way ANOVA test using the SPSS 17.0 software (SPSS Inc., Chicago, IL, USA); * *p* < 0.05 or ** *p* < 0.01 were considered statistically significant.

## Figures and Tables

**Table 1:** Identification of the compounds in MHP.

Peak	*t*_R_ (min)	[M + H]^+^/[M + Na]^+^ (*m*/*z*)	[M − H]^−^ (*m*/*z*)	Formula	MS^2^ Ions (*m*/*z*)	Identification
1	13.38	−	137.0246	C_7_H_6_O_3_	118.7616, 108.8020, 91.9109	protocatechuic aldehyde
2	16.63	−	163.0401	C_9_H_8_O_3_	163.0300, 118.9575, 94.9441	*p*-coumaric acid
3	21.38	−	179.0350	C_9_H_8_O_4_	160.8768, 134.9199, 82.7767	caffeic acid
4	25.19	−	387.1655	C_20_H_20_O_8_	369.2120, 340.9894, 207.0837, 163.0436, 119.0394	ethyl rosmarinate
5	28.81	−	337.0923	C_16_H_18_O_8_	172.9905, 162.8953, 154.9699, 136.9224	*p*-coumaroyl quinic acid
6	35.79	−	514.3244	−	466.0504, 412.9174, 378.0275, 251.9538	unknown
7	47.24	588.4072	−	−	570.5060, 475.3133, 362.3077, 249.0771	unknown
8	49.10	−	537.1034	C_27_H_22_O_12_	493.1606, 383.1935, 313.1821, 295.0162, 202.9409	lithospermic acid
9	56.90	−	577.1559	C_27_H_30_O_14_	311.0969, 269.0196, 241.0736	apigenin-7-*O*-rutinoside
10	58.02	−	740.4911	−	717.5318, 693.6151, 603.9049, 536.1751	unknown
11	60.17	633.1774, 611.1957	−	C_28_H_34_O_15_	615.2299, 483.1602, 331.0648	hesperidin
12	61.90	631.1624, 609.1804	−	C_28_H_32_O_15_	463.1456, 447.0624, 301.0494, 286.0661, 191.0885	diosmin
13	66.44	−	359.0767	C_18_H_16_O_8_	341.1331, 160.9566, 132.9843	rosmarinic acid
14	66.91	418.9493	−	C_20_H_18_O_10_	400.2057, 362.4225, 344.5225, 220.0517	salvianolic acid D
15	70.48	741.1419	−	C_36_H_30_O_16_	561.0992, 543.1505, 451.1976, 363.1163, 319.0901	salvianolic acid B
16	75.29	475.3232		−	457.2657, 317.2458, 249.2445, 167.2896	unknown
17	88.55	593.1849, 615.1667	−	C_28_H_32_O_14_	489.1708, 447.1117, 285.0378, 270.0775, 242.1294	linarin
18	90.61	797.2488	−	−	651.1746, 447.0908, 285.0492, 24.9773	unknown
19	107.36	361.0909	−	C_18_H_16_O_8_	346.0471, 328.0377, 300.1852, 213.0013, 150.7952	thymonin
